# Enrichment of *Porphyromonas gingivalis* in colonic mucosa-associated microbiota and its enhanced adhesion to epithelium in colorectal carcinogenesis: Insights from *in vivo* and clinical studies

**DOI:** 10.1371/journal.pone.0320383

**Published:** 2025-03-25

**Authors:** Shunya Motosugi, Naoki Takahashi, Shuhei Mineo, Keisuke Sato, Takahiro Tsuzuno, Yukari Aoki-Nonaka, Nao Nakajima, Kazuya Takahashi, Hiroki Sato, Haruna Miyazawa, Koji Taniguchi, Shuji Terai, Koichi Tabeta

**Affiliations:** 1 Division of Periodontology, Niigata University Graduate School of Medical and Dental Sciences, Niigata, Japan; 2 Division of Gastroenterology and Hepatology, Niigata University Graduate School of Medical and Dental Sciences, Niigata, Japan; 3 Clinical and Translational Research Center, Niigata University Medical and Dental Hospital, Niigata, Japan; 4 Department of Pathology, Faculty of Medicine and Graduate School of Medicine, Hokkaido University, Sapporo, Japan; University of the Pacific, UNITED STATES OF AMERICA

## Abstract

**Objectives:**

The oral–gut axis is believed to play a role in the pathogenesis of colorectal cancer (CRC). Previous studies have demonstrated the transmission of oral microbiota to the gut, disrupting gut microbial balance and creating a protumorigenic microenvironment conducive to CRC progression. *Fusobacterium nucleatum* is a putative periodontal pathogen recognized as a specific bacterium that promotes CRC development. However, the possible involvement of other periodontal pathogens in CRC is poorly understood. This study aimed to explore the effects of ingested periodontal pathogens on experimental CRC in mice and elucidate the underlying mechanisms.

**Methods:**

In this study, experimental colitis-induced CRC mouse models were used. The mice were orally administered periodontal pathogens (*Porphyromonas gingivalis* and *Prevotella intermedia*) three times a week during the experimental period. The CRC severity between the *P. gingivalis*-treated and *P. intermedia*-treated groups was compared. Lumen-associated microbiota (LAM) and mucosa-associated microbiota (MAM) were analyzed in both mouse and human samples. *In vitro* studies were conducted using intestinal epithelial cells to explore the possible mechanisms by which the periodontal pathogens affect the CRC development.

**Results:**

The *P. gingivalis*-treated group exhibited significantly increased CRC severity compared to the other groups among azoxymethane/dextran sodium sulfate (AOM/DSS)-induced mouse models. The LAM and MAM exhibited distinct bacterial compositions, and *P. gingivalis* was enriched more in MAM than in LAM. *In vitro* adhesion assays revealed that *P. gingivalis* had higher adhesive capacity to intestinal epithelial cells than *P. intermedia* and indicated the possible involvement of gingipains in such a capacity.

**Conclusion:**

*P. gingivalis* is enriched in MAM, and its subsequent adhesion to intestinal epithelial cells is potentially involved in the progression of CRC.

## Introduction

Periodontitis is a chronic inflammation that affects the supporting structures of the teeth; it is characterized by bacterial plaque accumulation, which leads to tissue destruction and potential tooth loss [1]. Numerous epidemiological and animal studies reported that periodontitis is associated with various extraoral inflammatory diseases, such as cardiovascular diseases, diabetes, and rheumatoid arthritis [2–4]. As the plausible causative mechanism of their biological associations, the leakage of pathogenic bacteria and proinflammatory cytokines originating from the periodontal lesion into the systemic circulation has been proposed [5]. Recent studies have also proposed the possible involvement of the oral–gut axis in systemic diseases as a new causative mechanism [6]. Research has shown that oral microbes, including periodontal pathogens, translocate to the gut, which results in the ectopic enrichment of oral bacteria in the gut and notable alteration of gut microbial balance [7]. The subsequent adverse effects have been demonstrated in gastrointestinal diseases, such as inflammatory bowel disease and colorectal cancer (CRC) [8,9].

CRC is a heterogeneous malignant disease; it is the third most diagnosed cancer worldwide and the second leading cause of mortality among patients with cancer [10]. The occurrence and progression of CRC are influenced by various environmental and genetic factors. As a key environmental factor, increased evidence has indicated the crucial role of specific bacteria in the pathogenesis of CRC [11]. *F. nucleatum* is a gram-negative anaerobic oral commensal and periodontal pathogen. It is recognized as a CRC-related bacterium. Clinical studies have reported that F. nucleatum is more abundant in cancerous than in noncancerous tissues and that its presence is correlated with poor prognosis in patients with CRC, particularly those in advanced stages [12]. In addition to the direct procarcinogenic properties of *F. nucleatum* to mucosal epithelium, a pivotal role of *F. nucleatum* on regulating tumor microenvironment, complex surrounding tumors consisting of various cellular and extracellular components, has been reported [13]. Despite the sufficient knowledge of the involvement of *F. nucleatum* in the pathogenesis of CRC, the involvement of other periodontal pathogens, including *P. gingivalis* and *P. intermedia*, remains poorly understood.

In addition to the oncogenic effects of specific bacteria, accumulating evidence indicates that dysbiosis of the gut microbiota is also associated with CRC development [14]. Most studies on gut microbiota focused on the use of fecal samples (referred to as lumen-associated microbiota [LAM]) owing to the convenience and noninvasiveness of fecal sampling. However, recent studies have reported another microbiota colonizing on the intestinal mucosa (referred to as mucosa-associated microbiota [MAM]) that exhibit distinct compositions from LAM [15–17]. From the biogeographic viewpoint, the microorganisms in MAM are more likely to directly interact with the intestinal epithelium than those in LAM, indicating the pivotal role of the former in the tumor microenvironment of CRC [18–20]. Nevertheless, the possible association of the periodontal pathogens of MAM with CRC development remains to be elucidated. Therefore, this study aimed to investigate the involvement of periodontal pathogens in CRC and explore the biological mechanism highlighting the MAM and LAM.

## Materials and Methods

### Mice

All the animal experiments were approved by the Committee for the Care and Use of Laboratory Animals of Niigata University (approval numbers: SA01272 and SA01375) and conducted in accordance with the Regulations and Guidelines for the Scientific and Ethical Care and Use of Laboratory Animals of the Science Council of Japan. Specific pathogen-free male C57BL/6 mice (6–8 weeks old) were obtained from Japan SLC, Inc. (Shizuoka, Japan). They were maintained in a specific sterile colony under completely controlled conditions (12-hour light/dark cycle with lighting at 8:00 am) and allowed access to a commercial diet and water *ad libitum*. As a procedure to alleviate suffering, cervical dislocation was performed following euthanasia by CO2 inhalation. Additionally, as a humane endpoint, euthanasia was carried out if a weight loss of more than 20% was observed within 7 days. Additionally, this study complies with the ARRIVE guidelines, ensuring rigorous and ethical reporting of all animal research procedures.

### AOM-DSS-induced CRC in mice

In this study, AOM/DSS (azoxymethane/dextran sodium sulfate)-induced experimental CRC models were used as previously described [21]. After a 1-week adaptation period, the mice were randomly allocated to three groups: sham group (*n* =  8), *P. g*-treated group (*n* =  9), and *P. i*-treated group (*n* =  8). The sample size for each group was determined based on a power analysis to detect a significant difference in tumor development between groups, with an expected effect size of 0.8, a two-sided significance level (alpha) of 0.05, and a desired power of 80%. Previous studies on CRC models in mice have demonstrated similar effect sizes with comparable group sizes, supporting the adequacy of our chosen sample size to ensure statistically meaningful results. The mice in all groups were intraperitoneally injected with AOM (10 mg/kg; Wako, Osaka, Japan) at the start day of the experiment. Then, they were given drinking water containing 2.5% DSS (36,000–50000 MW, MP Biomedical) for 1 week, followed by DSS-free drinking water for 2 weeks. The DSS treatment cycle was repeated three times. The body weights of the mice were monitored daily. At 9 weeks, the mice were sacrificed for sample collection. Their whole intestines were immediately removed and opened longitudinally. The number of visible tumors on the colorectal surface was counted with the naked eyes. For microbiota analysis in mice, LAM was derived from fecal samples, and MAM was obtained by swabbing the polyps and surrounding mucosal areas.

### Bacterial cultures and pathogen administration

Two putative periodontal pathogens, namely, *P. gingivalis* strain W83 and *P.* *intermedia* strain ATCC25611, were cultured as previously described [22]. They were cultivated in a modified Gifu Anaerobic Medium Broth (Nissui, Tokyo, Japan) using AnaeroPack™ (Mitsubishi Gas Chemical Co., Inc., Tokyo, Japan) and stored in an anaerobic jar (Becton Dickinson Microbiology System, Cockeysville, MD, USA) at a temperature of 37°C. By generating a standard curve relating the plate spread colony forming unit (CFU) and optical density (OD) at 600 nm, the OD measurement was used to estimate the number of cultured bacteria. After calibrating the number of bacteria by adjusting the OD value, the bacterial suspension was centrifuged at 3,000 rpm for 20 min. Then, the supernatant was discarded, and bacterial pellets were suspended in phosphate-buffered saline (PBS) with 2% carboxymethyl cellulose for *in vivo* experiments. Subsequently, 100 uL of the suspension at a concentration of 10^9^ CFU/mL of live bacteria was orally administered three times a week throughout the experimental period according to the protocol [23].The sham group was administered the same volume of vehicle as a control. Mice with severe weight loss exceeding 20% of baseline body weight during the study, or any signs of distress that could not be alleviated by standard procedures, were excluded from the study.

### Histological staining of intestinal tissues

For histochemical staining, the harvested intestinal tissues were rinsed with PBS, fixed overnight in 10% phosphate-buffered formalin solution, and then embedded in paraffin. The paraffin sections were cut and dewaxed before being stained with hematoxylin and eosin.

For immunohistochemistry, the sections were incubated with anti-β-catenin antibody (1:800, Rabbit-polyclonal, Proteintech, Rosemont, IL, USA) and PCNA antibody (1:800, Rabbit-polyclonal, Proteintech) overnight. Immunoreactivity was detected using the ImmPRESS® HRP Horse Anti-Rabbit IgG Polymer Kit (Vector Laboratories, Inc., Burlingame, CA, USA) and a secondary antibody-labeled polymer method. Counterstaining was performed using hematoxylin. Subsequently, the sections were imaged via microscopy (Biozero BZ-8000; Keyence Corporation, Osaka, Japan) and quantified using the Image J software.

### Total DNA extraction and sequencing of the 16S rRNA gene

Bacterial DNA from mice samples was extracted using the DNeasy Blood and Tissue Kit (Qiagen, Venlo, Netherlands) according to the manufacturer’s protocol. The extracted DNA was amplified using the bacterial 16S rDNA PCR Kit (Takara Bio Inc., Shiga, Japan). After confirming the specific amplification via agarose gel electrophoresis, the bacterial 16S rDNA band was excised from the gel and purified using the QIAquick PCR Purification Kit (Qiagen) according to the manufacturer’s protocol. The gel-purified DNA was quantified using NanoDrop (Thermo Scientific, Waltham, MA, USA). Bioengineering Lab. Co., Ltd. (Kanagawa, Japan) performed 16S ribosomal RNA gene sequencing. Briefly, the amplicon sequence library was prepared via two-tailed PCR. To amplify both the V3 and V4 regions of the 16S ribosomal RNA, the first PCR was conducted using the following primers: forward primer 5′ACACTCTTTCCCTACACGACGCTCTTCCGATCT-NNNNN-CCTACGGGNGGCWGCAG; reverse primer 5′ GTGACTGGAGTTCAGACGTGTGCTCTTCCGATCT-NNNNN-GACTACHVGGGTATCTAATCC). The thermal conditions were 94°C for 2 min, followed by 98°C for 10 s, 55°C for 30 s, and then 78°C for 30 s, with a final extension at 68°C for 7 min. Purification was performed using AMPureXP (Beckman Coulter, Brea, CA, USA), and the primers were removed. To attach the necessary adapter sequences and unique dual index for library preparation, the second PCR was performed using the following primers: forward primer 5′-AATGATACGGCGACCACCGAGATCTACAC TATAGCCTTCGTCGGCAGCGTC-3’; reverse primer5’-CAAGCAGAAGACGGCATACGAGAT CTAGTACG GTCTCGTGGGCTCGG-3.’ The thermal conditions were 94°C for 2 min, followed by 94°C for 30 s, 60°C for 30 s, and 72°C for 30 s, with a final extension at 72°C for 5 min. The indexed libraries were cleaned and analyzed using the Fragment Analyzer system and the dsDNA 915 Reagent Kit (Advanced Analytical Technologies, Ames, IA, USA). The prepared libraries were utilized for paired-end sequencing using MiSeq v3 reagents and 2 ×  300-bp reads on MiSeq (Illumina, San Diego, CA, USA).

### Microbiome analysis

Sequence data were processed using the QIIME2 platform (ver. 2022.8) for microbiome analysis. After being denoised using the QIIME2 DADA2 plugin, the sequences were produced into amplicon sequence variants (ASVs). The ASVs were assigned to the database using the feature classifier plugin, and operational taxonomic units were defined based on 97% similarity clustering using QIIME2 with default parameters. Bacterial taxonomy assignment was performed using the Greengenes (ver. 13_8) database. α- and β-diversities (microbial diversities between samples) were analyzed using the QIIME2 diversity plugin with default parameters. The relative abundance of the microbiota composition at the phylum and family levels of each sample was calculated and visualized. Linear discriminant analysis effect size (LEfSe) analysis was conducted using the LEfSe package (ver1.0.8). Specifically, the nonparametric factorial Kruskal–Wallis and Wilcoxon rank-sum tests were employed to identify differences, and linear discriminant analysis (LDA) was further conducted to evaluate the microbial effects for each group.

### Conventional PCR and gel electrophoresis

In this study, conventional PCR was performed using the Veriti PCR System (Applied Biosystems, Carlsbad, CA, USA) to detect bacteria in the mice samples. Amplification was conducted with predenaturation at 95°C for 30 s, followed by 40 cycles of 95°C for 10 s and 60°C for 30 s, using specific primers for *P. gingivalis* (forward primer 5’-AGGCAGCTTGCCATACTGCG-3’ and reverse primer 5’-ACTGTTAGCAACTACCGATGT-3’) and *P. intermedia* (forward primer 5’-CGTGGACCAAAGATTCATCGGTGGA-3’ and reverse primer 5’-CCGCTTTACTCCCCAACAAA-3’). PCR products were run on 1.5% agarose gels and visualized using SYBR® Safe DNA (Invitrogen Corporation, Carlsbad, CA, USA).

### Human specimen sampling

This study was conducted in accordance with the principles of the Declaration of Helsinki, received approval from the Ethics Committee of Niigata University Medical and Dental Hospital (Approval No: 2021-0229), and was conducted over an experimental period from January 17, 2022, to March 31, 2023. Informed consent was obtained in writing from all subjects involved in the study. Written consent was also obtained from the patients for publication of this paper. Additionally, this study complies with the STROBE guidelines, ensuring transparency and comprehensive detail in the reporting of our observational research.

A total of 20 patients who underwent endoscopic mucosal resection of colorectal lesions at the Division of Gastroenterology and Hepatology, Graduate School of Medical and Dental Sciences, Niigata University, were enrolled in this study. Inclusion criteria included patients aged 20 years or older, with confirmed colorectal lesions suitable for endoscopic mucosal resection, and with no history of systemic inflammatory diseases. Oral samples (saliva and subgingival dental plaque) and intestinal samples (feces and swab of intestinal mucosa) were collected. The exclusion criterion was consumption of antibiotics within 3 months before the study initiation. For saliva collection, the patients were instructed to spit into a sterile Falcon tube for 5 min. Two sterile paper points were inserted into the gingival sulcus for 10 s to collect subgingival dental plaque samples. The MAM was obtained by swabbing the surface of the polyp. All samples were immediately stored at − 80°C after collection. The clinical parameters and characteristics of the study participants are shown in Table 1.

**Table 1:** Clinical parameters and characteristics.

	(N = 20)
Remaining teeth	21.95 ± 8.04
Plaque control record	46.08 ± 25.07
Mean probing depth	2.935 ± 0.48
Bleeding on probing	15.6 ± 14.8
Deepest probing depth	5.75 ± 1.59

### DNA extraction from human clinical specimens

Samples suspended in preservation solution were transferred to EZ-Beads (Promega, Madison, WI, USA) and homogenized for 3 min. Then, the supernatant was boiled for 5 min. DNA was extracted using GeneFind V2 (Beckman Coulter) according to the manufacturer’s protocol.

### Bacterial detection using real-time PCR

For human clinical specimens, real-time PCR was performed with the extracted DNA at a final volume of 20 μL per reaction using PowerUp™ SYBR™ Green Maser Mix (Applied Biosystems) on the QuantStudio 1 PCR system (Applied Biosystems). Amplification was conducted with predenaturation at 95°C for 30 s, followed by 40 cycles of 95°C for 10 s and 60°C for 30 s, using specific primers for *P. gingivalis* (forward primer 5’-AGGCAGCTTGCCATACTGCG-3’ and reverse primer 5’-ACTGTTAGCAACTACCGATGT-3’) and *P. intermedia* (forward primer 5’-CGTGGACCAAAGATTCATCGGTGGA-3’ and reverse primer 5’-CCGCTTTACTCCCCAACAAA-3’). In this study, we considered a cutoff Ct of < 38 as indicating positive bacterial detection in clinical specimens referred to previous publication [23].

### Cell culture

The human intestinal epithelial cell line Caco-2 was provided by RIKEN BioResource Center (Tsukuba, Japan). The Caco-2 cells were cultured in Dulbecco’s Modified Eagle Medium supplemented with 10% fetal bovine serum, 100-U/mL penicillin, and 100-µg/mL streptomycin.

### Bacterial adhesion assay

An adhesion assay was conducted using a standard bacterial adhesion assay [24]. Briefly, the Caco-2 cells were cultured in subconfluently and then incubated with the indicated bacterial suspension at a multiplicity of infection of 100 for 2 h. OD measurement was performed to estimate and calibrate the number of bacteria for experiment. The bacterial -treated plate was washed three times with cold PBS, and the cells were removed from the plate using trypsin EDTA. To determine the number of CFU of the adhered bacterial cells, serial dilutions of lysates were plated on blood agar plates (Becton, Dickinson and Company, Franklin Lakes, NJ, USA).

### Statistical analysis

All data were expressed as mean ±  standard deviation. Statistical analyses were conducted using GraphPad Prism (GraphPad Software, San Diego, CA, USA). The Mann–Whitney U test was employed for two-group comparisons, whereas one-way analysis of variance with Tukey’s post hoc test was used for multiple-group comparisons. Meanwhile, Fisher’s exact test was employed for clinical samples. A P-value <  0.05 was considered statistically significant.

## Results

### Oral administration of *P. gingivalis* aggravates colitis-associated colorectal cancer in experimental mouse models

**Fig 1:**
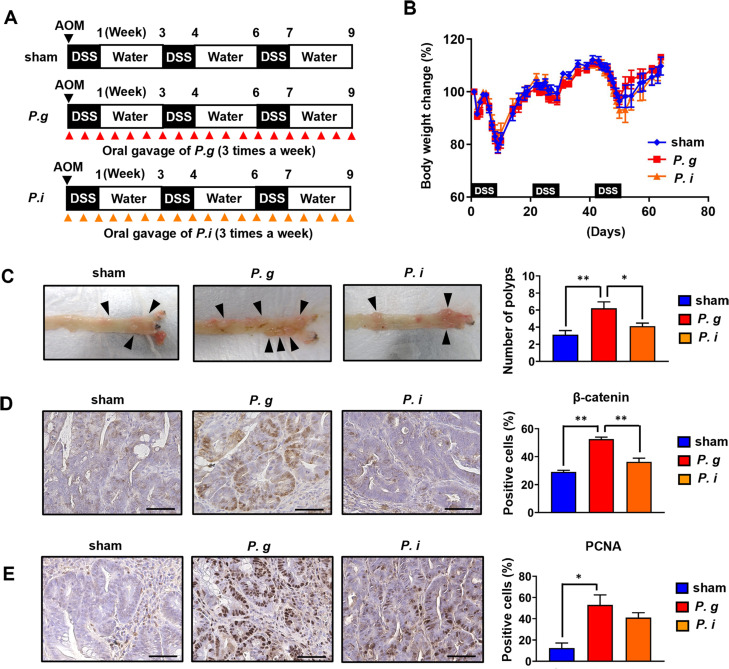
Oral administration of *P. gingivalis* exacerbates colitis-associated colorectal cancer in experimental mouse models. (A) Experimental design of bacterial treatment in the AOM-DDS model. Sham: control group treated with vehicle only. (B) Body weight change in all the groups with or without periodontal pathogen administration. Data were expressed as percentage of body weight at baseline (*n* =  8–9 in each group). (C) Representative macroscopic images of colonic polyps (left panel). The arrowheads indicate the colonic polyps. Quantification of tumor numbers (right panel) (*n* =  8–9 in each group). (D) β-catenin immunohistochemistry staining; scale bars: 50 μm (left panel). Quantification of the percentage of β-catenin-positive cells (right panel) (*n* =  3 in each group). (E) PCNA immunohistochemistry staining; scale bars: 50 μm (left panel). Quantification of the percentage of PCNA-positive cells (right panel) (*n* =  3 in each group). The asterisk denotes statistical significance ( *P <  0.05; ** P <  0.01). Scale bars: 50 μm.

To investigate the carcinogenic effect of periodontal pathogens, *P. gingivalis* and *P. intermedia* were orally administrated to AOM-DSS-induced CRC mouse models (Fig. 1A). The body weights of the mice were monitored throughout the experimental period, and temporary weight loss due to AOM and DSS administration was observed; however, there were no differences in weight change between the groups (Fig. 1B). Morphologically, polyps were found on the mucosal surface of the colon and were more abundant in the *P. g*-treated group than in the sham and *P. i*-treated groups (Fig. 1C). Furthermore, histological evaluation revealed that the number of positive cells of both β-catenin and PCNA, which are colorectal carcinogenesis-related proteins, in the intestinal epithelium was significantly higher in the *P. g*-treated group than in the sham and *P. i*-treated groups (Fig. 1D, E). Taken together, these findings indicate that oral administration of *P. gingivalis* has carcinogenic potency in experimental AOM-DSS-induced CRC in mice.

### Microbiome analysis revealed distinct profiles of the LAM and MAM, with possible enrichment of orally administered *P. gingivalis* in the gut

**Fig 2:**
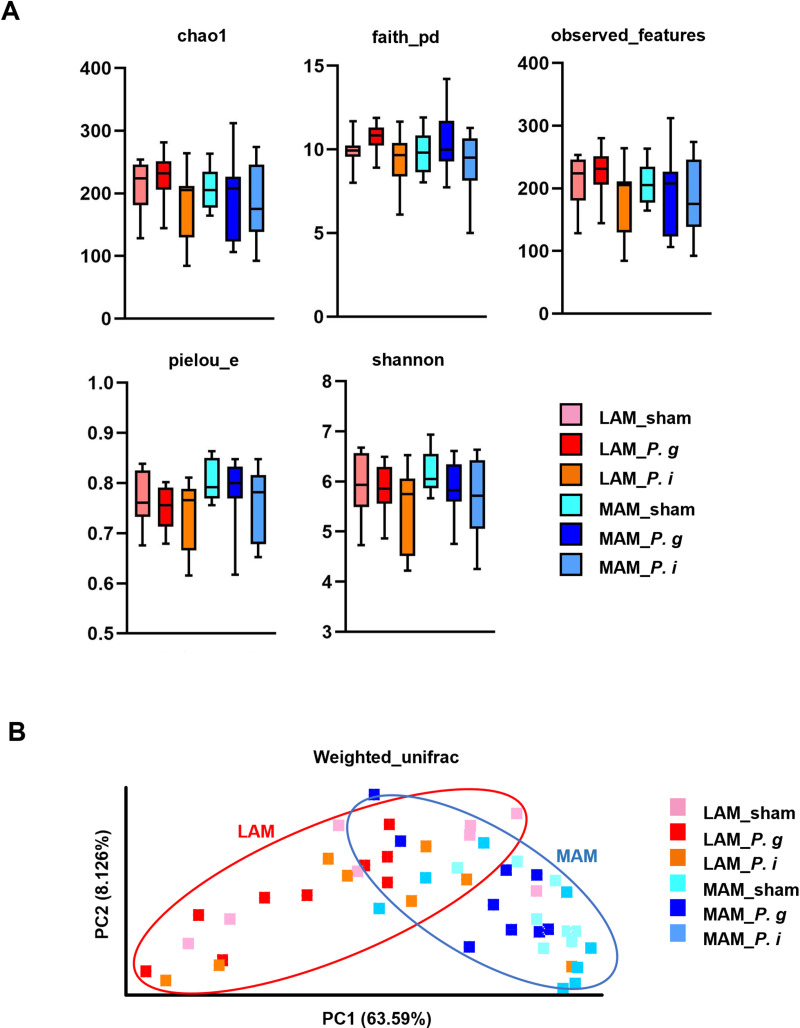
The bacterial diversity of the samples was segregated between LAM and MAM. (A) Alpha diversity of the microbiome of each sample. The box and whiskers indicate the mean, standard deviation (box), and min–max (whisker) (*n* =  8–9 in each group). One-way ANOVA was employed for the test of difference and Tukey for the post hoc test. (B) PCoA plot of beta diversity based on weighted UniFrac distance for each sample.

**Fig 3:**
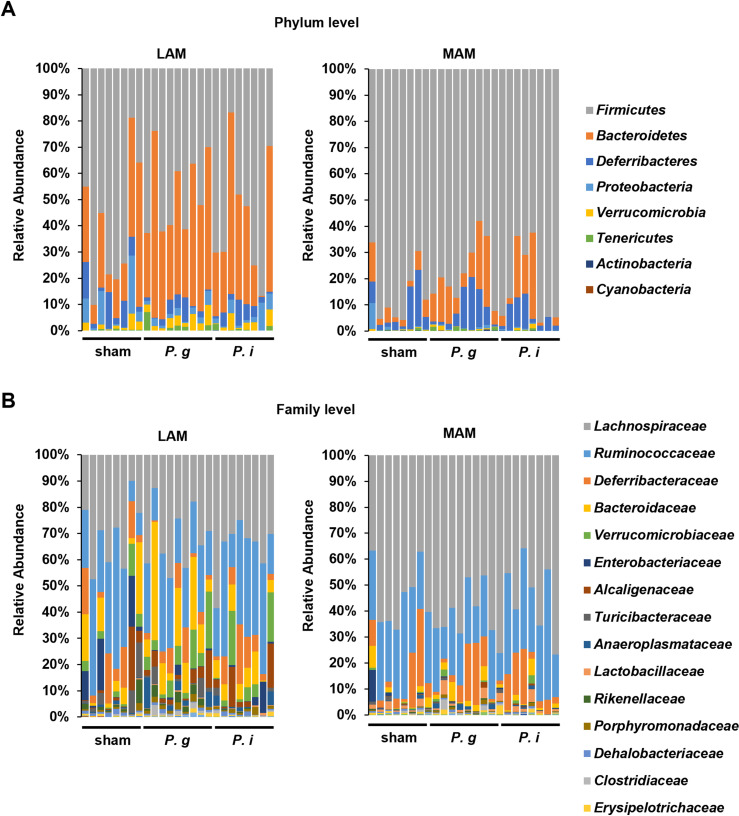
LAM and MAM showed distinct trends in the microbiota composition. (A) Bacterial composition in LAM and MAM at the phylum level (*n* =  8–9 in each group). (B) Bacterial composition in the LAM and MAM at the family level (*n* =  8–9 in each group).

**Fig 4:**
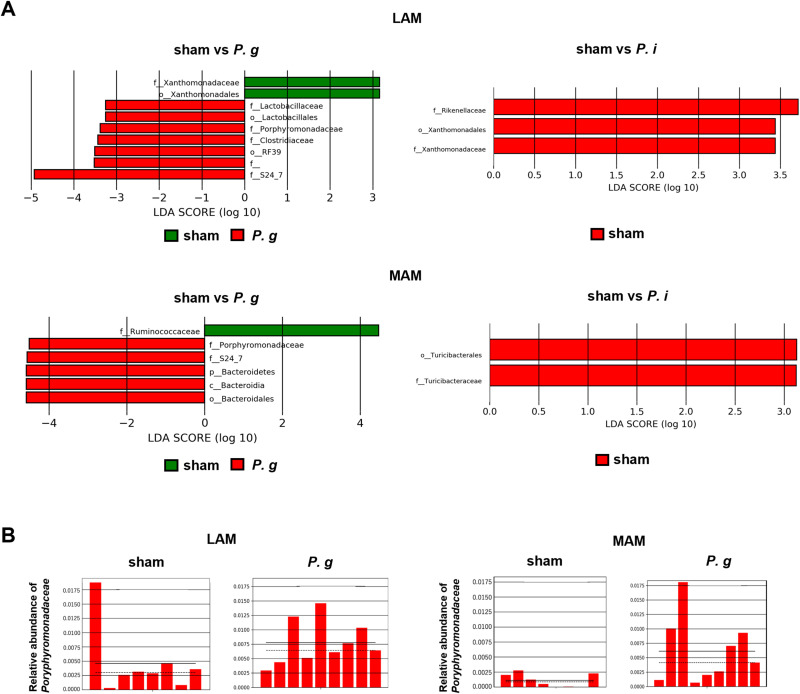
High abundance of *Porphyromonadaceae* in *P. g*-treated LAM and MAM. (A) Linear discriminant analysis (LDA) effect size (LEfSe) plot of the indicated comparison pairs in LAM (upper panel) and MAM (lower panel). The horizontal red and green bars represent the effect size for each bacterium. The LDA scores represent the log10-transformed LDA score. Negative as well as positive values denote increased abundance compared with the other group. (B) Relative abundance of *Porphyromonadaceae* in the indicated groups. Each red bar of the histograms represents the relative abundance of *Porphyromonadaceae*. The solid and dotted horizontal black lines indicate the mean and median relative abundance values for each group, respectively.

As alterations in the gut microbiota are associated with CRC, we conducted microbiome analysis with a focus on the LAM and MAM. For the α-diversity analysis, each diversity index exhibited no significant differences among the groups (Fig. 2A). For the β-diversity analysis, the PCoA plot using unweighted UniFrac distance showed the tendency to segregate between the LAM and MAM (Fig. 2B). Similarly, the distinct tendency of bacterial compositions was observed between the LAM and MAM at the phylum and family levels (Fig. 3A, B). Subsequently, we conducted LEfSe analysis to further characterize the taxonomic differences among the groups; the results showed that the *P. g*-treated groups exhibited a significantly higher relative abundance of the family *Porphyromonadaceae*, to which *P. gingivalis* belongs, compared to the sham group. (Fig. 4A). No differences were observed in the abundance of the family *Prevotellaceae* which *P. intermedia* belongs to between the sham and *P. i*- treated groups. Relative abundance of *Porphyromonadaceae* in both the LAM and MAM was increased by the *P. gingivalis* treatment, indicating possible enrichment of orally administered *P. gingivalis* in the gut.

### *P. gingivalis* exhibited higher presence in the MAM than in the LAM

**Fig 5:**
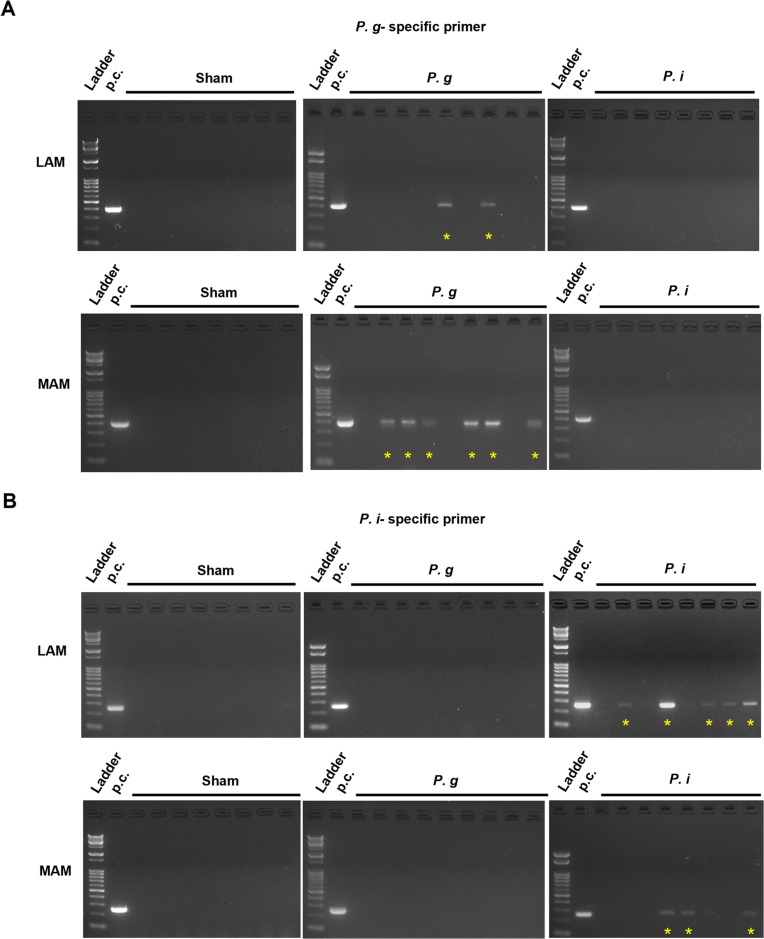
*P. gingivalis* exhibited a tendency to be detected more in MAM than in LAM. (A) Agarose gel electrophoresis of the conventional PCR used to detect *P. gingivalis* in the LAM (upper panel) and MAM (lower panel). (B) Agarose gel electrophoresis to detect *P. intermedia* in the LAM (upper panel) and MAM (lower panel). The yellow asterisk indicates the positive band, and p.c. indicates positive control.

**Fig 6:**
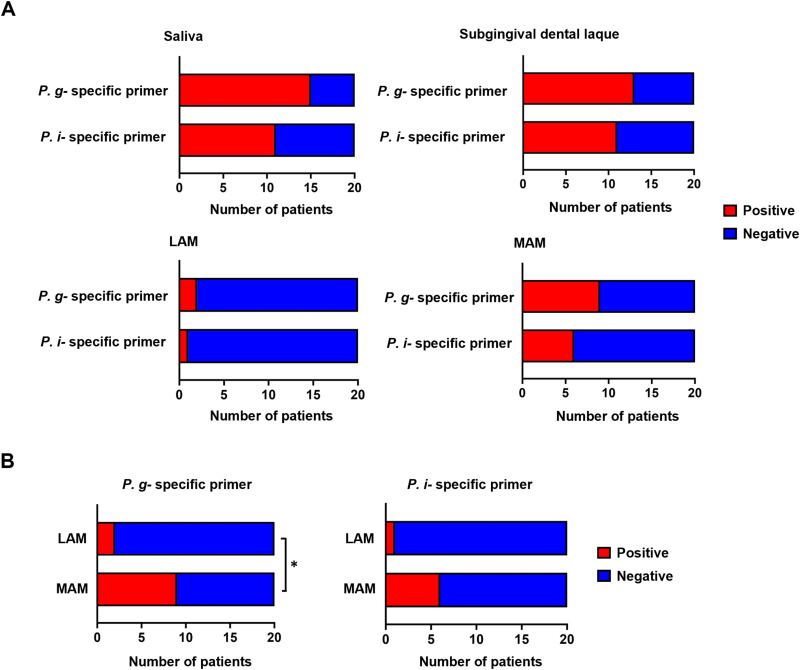
*P. gingivalis* was significantly enriched in MAM in the clinical samples. (A) Number of patients who were positive for *P. gingivalis* or *P. intermedia* in the indicated samples (*n* =  20 in total). (B) Comparison of patients positive for *P. gingivalis* (left panel) and *P. intermedia* (right panel) in the LAM and MAM (*n* =  20 in total). The asterisk represents statistical significance (*P <  0.05).

To confirm the enrichment of orally administered bacteria in the MAM and LAM, we conducted PCR analysis using specific primers for *P. gingivalis* and *P. intermedia*. The conventional PCR analysis conducted in this study using mice samples revealed that *P. gingivalis* was present in both the LAM and MAM and was more abundant in the latter than in the former (MAM: 6 out of 9 samples, LAM: 2 out of 9 samples) (Fig. 5A). An opposite trend was observed for *P. intermedia*, being more abundant in the LAM than in the MAM (LAM: 5 out of 8 samples, MAM: 3 out of 8 samples) (Fig. 5B). We also performed similar quantification using clinical samples obtained from human patients with colonic polyps. Both *P. gingivalis* and *P. intermedia* were detectable in all types of samples, such as saliva, subgingival dental plaque, feces (referred to as LAM), and swab of intestinal mucosa (referred to as MAM) via real-time PCR (Fig. 6A). Interestingly, *P. gingivalis* was found to be more abundant in the MAM than in the LAM (Fig. 6B), indicating the extensive enrichment of *P. gingivalis* in the surface of the intestinal mucosa.

### *P. gingivalis* showed higher adhesion capacity to intestinal epithelial cells than *P. intermedia*

**Fig 7:**
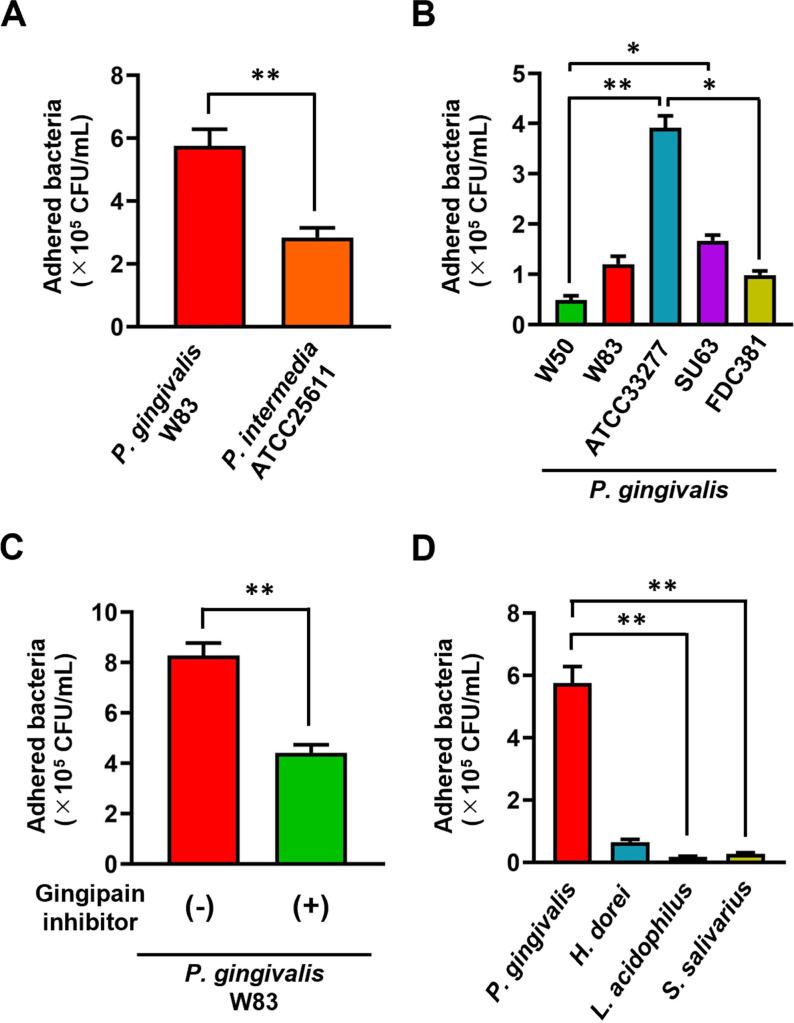
The higher adhesion capacity of *P. gingivalis* to intestinal epithelial cells compared with *P. intermedia.* (A) Comparison between *P. gingivalis* and *P. intermedia* in terms of adhesion capacity to Caco2 cells (*n* =  6 in each group). (B) Comparison of the adhesion capacity among the *P. gingivalis* strains (*n* =  6 in each group). (C) Comparison of the adhesion capacity of *P. gingivalis* with or without gingipain inhibitors (*n* =  6 in each group). (D) Comparison with intestinal (*H. dorei* and *L. acidophilus*) and oral commensal bacteria (*S. salivarius*) (*n* =  6 in each group). The asterisk represents statistical significance ( * P <  0.05; ** P <  0.01).

Subsequently, we explored the interaction of the periodontal pathogens enriched near the intestinal mucosal surface with the host epithelial cells. An *in vitro* adhesion assay using human intestinal epithelial cells clearly revealed the higher adhesion capacity of *P. gingivalis* to the cells than *P. intermedia* (Fig. 7A). Furthermore, the analysis of different strains of *P. gingivalis* revealed the strain-dependent adhesive capacity to the intestinal epithelial cells (Fig. 7B). Moreover, we observed that the pretreatment of *P. gingivalis* with specific inhibitors to gingipain, a major virulence factor produced by *P. gingivalis*, significantly decreased its adhesion capacity to the cells (Fig. 7C). *P. gingivalis* exhibited significantly higher adhesion ability compared to intestinal and oral commensal bacteria (Fig. 7D).

## Discussion

This study demonstrates that ingested *P. gingivalis*, a putative periodontal pathogen, aggravates CRC in an AOM/DSS mouse model. Our animal study and clinical examination revealed that *P. gingivalis* was more abundant in the MAM than in the LAM. Furthermore, our *in vitro* study showed that *P. gingivalis* exhibited higher adhesion capacity to intestinal epithelial cells than *P. intermedia*. These results indicate that *P. gingivalis* is involved in the pathogenesis of CRC owing to its high MAM translocation and high adherence to the intestinal epithelium.

In this study, AOM/DSS-induced mouse models were used to investigate CRC (Fig. 1). Various CRC mouse models, including genetically derived and chemically induced models, are available for CRC research. Genetically engineered Apc^Min/+^ mice exhibit mutation at the adenomatous polyposis coli (Apc) tumor suppressor gene and shows consequent predisposition to multiple intestinal neoplasia [25]. Wang *et al.* recently reported that oral gavage of *P. gingivalis* increased the tumor count and volume in the Apc^Min/+^ mouse model [26]. In addition, they demonstrated that *P. gingivalis* promoted CRC via NLRP3 inflammasome activation *in vitro* and *in vivo*. Among the chemically induced CRC models, the combination with the AOM and DSS is the most common method and representative and robust polyp induction is observed [21]. AOM is a colonic epithelial cell-specific carcinogen that causes the formation of preneoplastic lesions by DNA damage. DSS is a chemical that induces colonic inflammation, which promotes the development of the initiated neoplastic cells into colorectal tumors. Recent studies using the AOM-DSS model have demonstrated that *P. gingivalis* administration exacerbates colorectal cancer severity by modulating specialized immune cells in the intestine, such as invariant natural killer T cells [27]. Previously, we have reported that *P. gingivalis* administration worsens colitis severity in a DSS-induced model [22]. Taken together, these findings suggest that the synergistic effects of *P. gingivalis*-mediated inflammation and immune modulation may contribute to the progression of colorectal cancer.

Our microbiota analysis using mice samples revealed that the LAM and MAM exhibited distinct bacterial profiles, which is consistent with previous studies (Figs. 23–4) [16,28,29]. Miyauchi *et al.* conducted a comparative analysis between LAM and MAM in healthy volunteers and found clear differences in both UniFrac and principal coordinate analysis [16]. Clavenna *et al.* recently reported the definite differences in the bacterial composition and diversity between LAM and MAM in patients with colonic polyp [18]. Moreover, they found distinct signatures of MAM between low- and high-grade dysplastic colon polyps, indicating alterations of MAM with a tumor-stage-specific manner. Considering the biogeographic adjacency of MAM to the intestinal epithelium, MAM has strong impacts on the initiation and development of colonic tumors. Therefore, MAM could be a potential diagnostic and therapeutic target of CRC.

In addition to the distinction between MAM and LAM, we examined the enrichment of *P. gingivalis* in MAM using PCR methods in both mice and human samples (Figs. 5 and 6). This could be explained by the high translocation of *P. gingivalis* to intestinal mucosal surfaces owing to its specific virulence factors. The intestinal epithelium is covered by a thick mucus layer that functions as an intestinal barrier separating lumen bacteria from the intestinal mucosa. The mucus layer is composed of highly glycosylated mucin proteins that form a gel-like structure overlying the intestinal epithelium. Mucin 2 (MUC2) synthesized by goblet cells is the most abundant mucin protein in the small and large intestines [30]. Gingipains, a group of complex arginine- or lysine-specific cysteine proteinases, have been recognized as a major virulence factor of P. gingivalis [31]. A previous study reported that RgpB, a type of gingipains secreted by *P. gingivalis*, exhibited the ability to cleave MUC2 at a specific site, resulting in the disruption of the MUC2 polymeric framework [32]. One of the factors contributing to the intestinal barrier is antimicrobial peptides, which prevent microorganisms from reaching the intestinal mucosa. Through mass spectrometry analysis, Carlisle *et al.* found that *P. gingivalis* culture supernatants fully or partially degrade human α- and β-defensins, which are major antimicrobial peptides secreted within the intestinal mucosa [33]. In addition, Maisetta *et al.* reported the degradation of human β-defensin by gingipains and its subsequent reduction *in vitro* [34]. Taken together, these observations suggest that the manipulation of the intestinal mucosal barrier by *P. gingivalis* allows its translocation and enrichment to the mucosal surface.

In our *in vitro* study, we found that *P. gingivalis* exhibited higher adhesion capacity to intestinal epithelial cells than other periodontal pathogens (Fig. 7). Furthermore, we exhibited the strain- and gingipain-dependent adhesive capacities of *P. gingivalis* to the cells. Bacterial adherence/invasion to the host cell is an important initial phase for the successful establishment of infection and subsequent cellular response [35]. The high adhesion/invasion capacities of *P. gingivalis* to oral epithelial cells have been extensively reported, with particular emphasis on the crucial roles of fimbriae and gingipains in the adhesion/invasion processes [36–38]. *P. gingivalis* fimbriae are classified into six types (types I to V and Ib) based on the fimA genes encoding FimA (a subunit of fimbriae), and the fimbria variations exhibit distinct adhesion properties [39]. We believe that the differences in adhesion profiles observed in this study were due to the strain-specific variations in the fimbria types. The involvement of gingipains in epithelial cell adhesion has also been reported. Chen *et al.* demonstrated that gingipains possess a catalytic domain and hemagglutinin/adhesin domains, which contribute to the adherence of *P. gingivalis* to gingival epithelial cells [40]. Moreover, Onoe *et al.* reported that the proteolytic function of gingipains is responsible for the maturation of fimbriae formation [41]. These findings likely explain the reduction of cell adhesion capacity by the gingipain-specific inhibitors in this study. Considering the recent publication reporting the presence of *P. gingivalis* in human intestinal tissues [10], further investigations are warranted to determine the impact of fimbriae and gingipains on intestinal epithelial adhesion and the subsequent cancer phenotypes.

This study has several limitations that need further investigation. First, this study did not include *F. nucleatum* as a comparison, as its association with CRC has already been well-documented in numerous studies. Rather than reiterating findings on *F. nucleatum*, we aimed to expand the focus to other periodontal pathogens, such as *P.* *gingivalis* and *P.* *intermedia*, to explore their potential role in CRC pathogenesis. However, including *F. nucleatum* as a positive control could have provided a useful comparative baseline. Second, the microbiota analysis in this study was limited to short-read sequencing, which did not achieve species-level identification. Therefore, we used qPCR to achieve species-level analysis for the two species of interest. A comprehensive metagenomic analysis would be necessary to provide broader species-level insights. Third, more comprehensive clinical studies are needed. In this study, the analysis was limited to patients with polyps, and future research should aim for a more extensive and large-scale approach. To obtain clearer insights, comparisons should include both healthy individuals and CRC patients, as well as evaluating variations across different CRC severity levels. Comparing bacterial enrichment in MAM between patients with and without polyps, as well as examining the effects of interventions such as periodontal treatment and oral care on the MAM flora, will deepen our understanding of the oral-gut connection in periodontal medicine. This understanding will provide new insights into the importance of maintaining healthy oral hygiene and the exploration of potential therapeutic interventions targeting the oral–gut microbial axis.

## Conclusions

*P. gingivalis* is enriched in MAM, and its subsequent adhesion to intestinal epithelial cells is potentially involved in the pathogenesis of CRC.

## Supporting Information

S1_RAW _images The original uncropped gel images for Fig 5.
(PDF)

## Abbreviations

AOM: azoxymethane
DSS: dextran sodium sulfate
*P. g*: *P. gingivalis*
*P. i*: *P. intermedia*
LAM: lumen-associated microbiota
MAM: mucosa-associated microbiota
