# Supplementary material for: Enrichment of Porphyromonas gingivalis in colonic mucosa-associated microbiota and its enhanced adhesion to epithelium in colorectal carcinogenesis: Insights from in vivo and clinical studies
Source: PLoS One. 2025 Mar 25;20(3):e0320383. doi: 10.1371/journal.pone.0320383 (PMC11936212; doi:10.1371/journal.pone.0320383)
Supplement: S1_RAW _images — The original uncropped gel images for Fig 5. (PDF) [file pone.0320383.s001.pdf]

P. g - specific primer

LAM

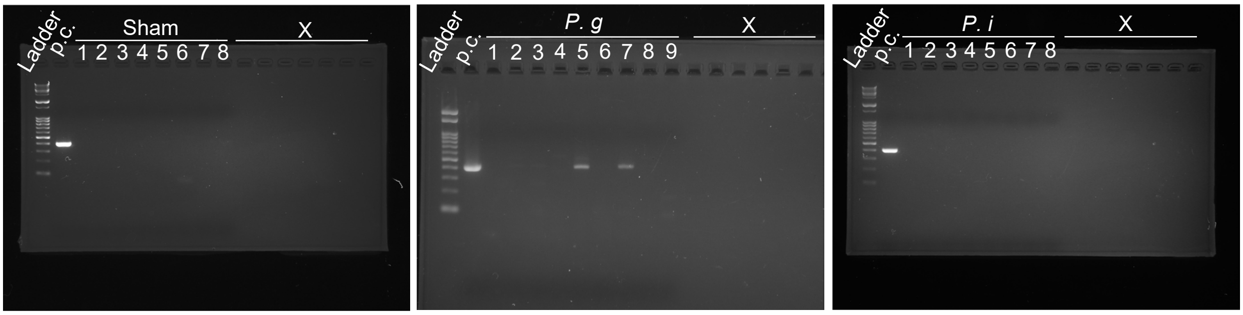

MAM

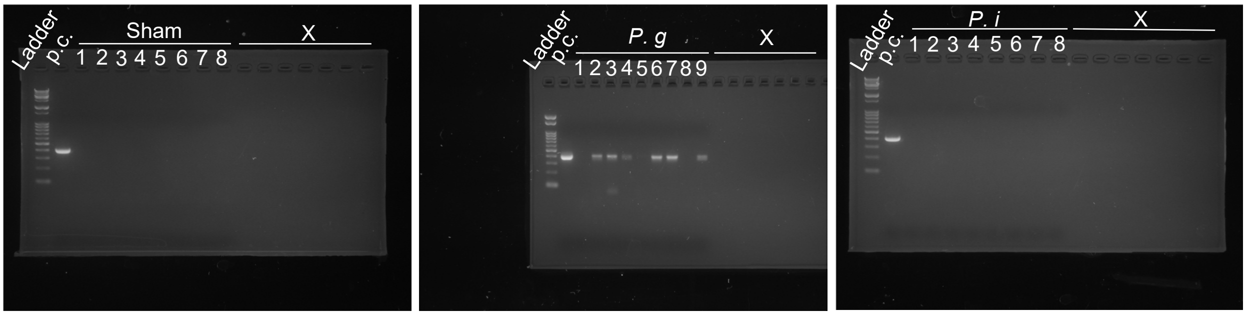

P. i - specific primer

LAM

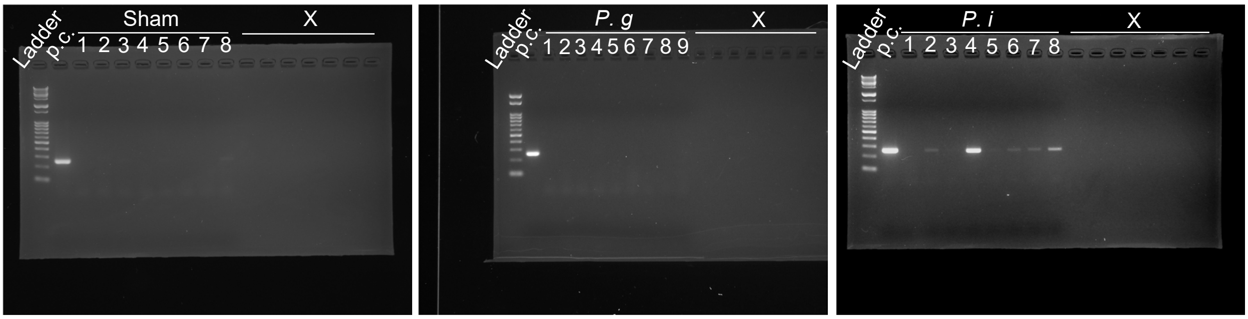

MAM

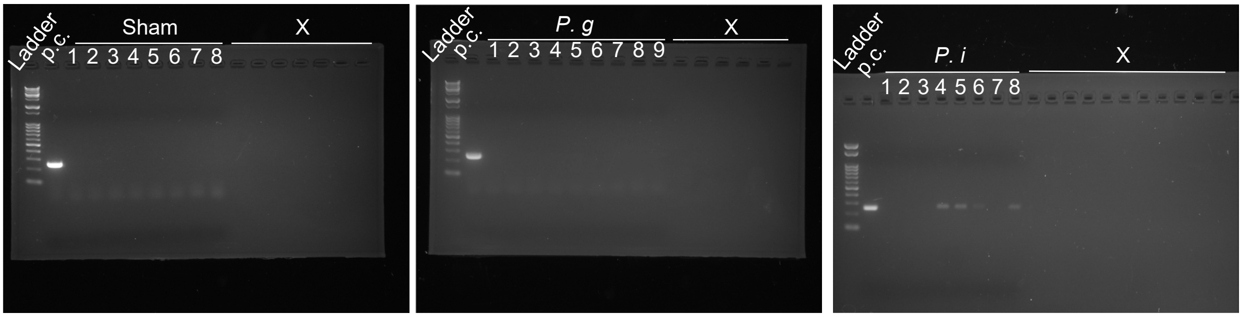

∗ All images were captured with a digital camera in dark box.
